# Clustering Complex Chronic Patients: A Cross-Sectional Community Study From the General Practitioner’s Perspective

**DOI:** 10.5334/ijic.5496

**Published:** 2021-04-19

**Authors:** Francisco Hernansanz Iglesias, Joan Carles Martori Cañas, Esther Limón Ramírez, Clara Alavedra Celada, Carles Blay Pueyo

**Affiliations:** 1 INSTITUT CATALA DE LA SALUT (ICS), EAP Nord, Sabadell, Spain; 2 University of Vic - University of Central Catalonia, Spain; 3 INSTITUT CATALA DE LA SALUT (ICS), EAP Mataró-7, Spain; 4 INSTITUT CATALA DE LA SALUT (ICS), EAP Ca N’Oriac, Spain; 5 University of Vic, Faculty of Medicine, Spain

**Keywords:** primary care, integrated care, complex chronic patient, patients’ complexity clusters, complex care needs, multimorbidity

## Abstract

**Objective:**

Characterize subgroups of Complex Chronic Patients (CCPs) with cluster analysis from the general practitioner’s perspective.

**Study design:**

Cross-sectional population-based study.

**Setting:**

Three Primary Care urban centres for a reference population of 43,647 inhabitants over 14 years old in Sabadell, Catalonia, Spain.

**Methods:**

Complexity is defined by the independent clinical judgment of general practitioners with the aid of complexity domains (both clinical and social). We used a Two-Step Cluster method to identify relevant subgroups of CCPs.

**Results:**

Three relevant subgroups were identified. The first one was mainly managed by primary care professionals, and 63% of its CCPs belonged to the high-risk stratum of the Adjusted Morbidity Groups (GMA). The second subgroup included younger patients than the other two clusters, and showed the highest ratios of social deprivation and severe mental disease; 48% of its CCPs belonged to the high-risk stratum of the GMA. A third cluster included patients who belonged to the high-risk stratum of the GMA. Their age was similar to that of the patients in the first cluster, but they showed the highest values in the following areas: (i) risk of admission; (ii) proportion of advanced chronic disease and limited-life prognosis; (iii) functional loss and (iv) geriatric syndromes, along with special uncertainty in decision-making and clinical management.

**Conclusions:**

Characterization of CCPs shows clearly distinct profiles of needs, which provides an improved epidemiological picture by identifying clusters of patients who are likely to benefit from targeted interventions.

## Introduction

### Background/rationale

In public health services, aging and a high prevalence of multiple diseases as age increases are currently the norm rather than the exception, and challenge the single-disease model that prevails in medical education, research and hospital care [1,2]. Individuals with multimorbidity do not show dominant combinations of conditions, and most clinical programs or guidelines for chronic disease management still focus on specific and single conditions. For these reasons, there is a growing concern that these programs may be less effective and even harmful for individuals with multimorbidity when compared to person-centred approaches [3]. In recent years, a new concept has been introduced, which is becoming increasingly common in primary care: the “complex chronic patient (CCP)” [4,5]. It encompasses a more holistic approach where not only health issues (multiple chronic conditions, mental health, medication-related problems) but also socio-economic, cultural and environmental factors play an essential role [6], thus reflecting person-specific factors that interfere with the usual provision of care and the provision of decision-making processes [7]. This group of patients has many care requirements that are also expensive, hence the current name of High-Need, High-Cost (HNHC) patients [8].

A common feature stands out from the literature review about the complexity construct [9,10,11,12,13,14]: the existence of dimensions other than multimorbidity that demonstrate the existence of social inequalities in health. Such dimensions include socioeconomic determinants of health (poverty, ethnic differences, low educational level and low social capital), culture-related issues (health literacy, lack of perceived multimorbidity), environmental issues (neighbourhood pollution, urban ghettos), patient behaviour (harmful habits, unhealthy diet) and their experience in the use of health services (accessibility, poor coverage, patient-practitioner interaction, etc.) [1]. Loeb et al. detected complexity in primary care if patients had an exacerbating factor—a medical illness, a mental disease, socioeconomic challenges, or a behaviour or trait (or some combination thereof)—that complicate care for chronic health conditions [15].

Complexity as defined by physicians only agrees moderately with traditional comorbidity algorithms [16]. Most current methods for measuring complexity are based on the pooling of weighted diagnoses, prior healthcare costs and use of resources. In spite of this, patient complexity is probably a multifaceted concept that is not always adequately taken into account by current multimorbidity groupers [17]. Addressing patients only on a cost basis, without taking into account personal characteristics and needs, may not identify them properly, and could be the reason why many programs are unsuccessful. Clustering chronic complex patients could identify subgroups with similar needs, thus allowing primary care patient panels to work with more specific and proactive approaches that would improve health outcomes while reducing costs.

The Chronicity Care and Prevention Program (PPAC) [18,19] by the Department of Health of Catalonia promoted the conceptualization, identification and integrated clinical management of complex chronic patients (CCPs). The allocation of patients in this category was based on the clinical judgment of their referent primary care professionals along with stratification strategies or risk groupers. However, so far there is no gold standard for an unambiguous identification. Catalonia also cooperates with the SUSTAIN Project (Sustainable Tailored Integrated Care for Older People in Europe) which aims to generate evidence on how to improve integrated care and apply and transfer the knowledge gained to inform and support policy-makers and decision-makers involved in integrated care [20]. In this initiative, health and social professionals work together and meet the patient and the caregiver at home, asking the patient what his/her personal goals are in terms of health and wellbeing and what his/her preferences would be concerning care options. They then validate the care plan, adjusting it to the user’s (and caregiver’s) needs and preferences.

There is a need to deepen the knowledge of clinical complexity from population-based perspectives in order to optimize public health policies.

### Objective

The aim of this study is to identify sub-populations of complex chronic patients who could benefit from targeted care management approaches.

## Theory and Methods

### Study design

Cross-sectional, population-based observational study.

### Study population

The present study was carried out in the town of Sabadell in the province Barcelona (Spain), with an approximate population of 207,444 inhabitants [21] (48.6% male; people over the age of 65: 18%; people under the age of 14: 16%). The study focuses on adult population cared for three urban primary care centres managed by the Institut Català de la Salut (ICS) (***Table 1***). These Primary Care Centres are partners of the SUSTAIN Project. They use and share electronic medical records for patient registration, daily clinical practice, morbidity recording and drug prescribing. It should be noted that the centres mentioned above care for a different number of patients who differ in their socioeconomic backgrounds according to the MEDEA deprivation index, an index validated in Spain and based on urban socioeconomic indicators as stated in the Spanish census [22]. The Medea index is calculated using five census-based socioeconomic indicators (percentages, by census tract): 1) unemployment rate, 2) manual workers, 3) temporary workers, 4) illiterate adults (or less than basic, compulsory education), and 5) school drop-outs among the under-16 population. The higher the Medea index, the worse the socioeconomic conditions are.

**Table 1:** Distribution and characteristics of the Primary Care services that participated in the study.


PRIMARY CARE CENTRE	POPULATION ≥14 YEARS OLD. REGISTERED*	SERVED*	%>75 YEARS OLD	MEDEA INDEX

NORD	13,700	84.04%	9.22%	1.56

CA N’ORIAC	17,198	82.17%	9.97%	1.41

CONCORDIA	12,749	82.57%	9.37%	0.49

Total	43,647			


* Medically served, which are less than those registered.

### Study subjects

The study analyses complexity in patients over age 14, registered in any of the three practices, and with a Clinical Risk Group (CRG) ≥ 5, which means that they suffer from at least one chronic dominant condition. Further details are provided in the variables section below.

### Data collection

In our National Health System, each patient is assigned to a general practitioner (GP), which means that these professionals are provided with a well-defined list of “their” patients. Registration in a general (primary care) practice is required to access health-care services and to obtain referrals to specialized care.

**Figure 1:**
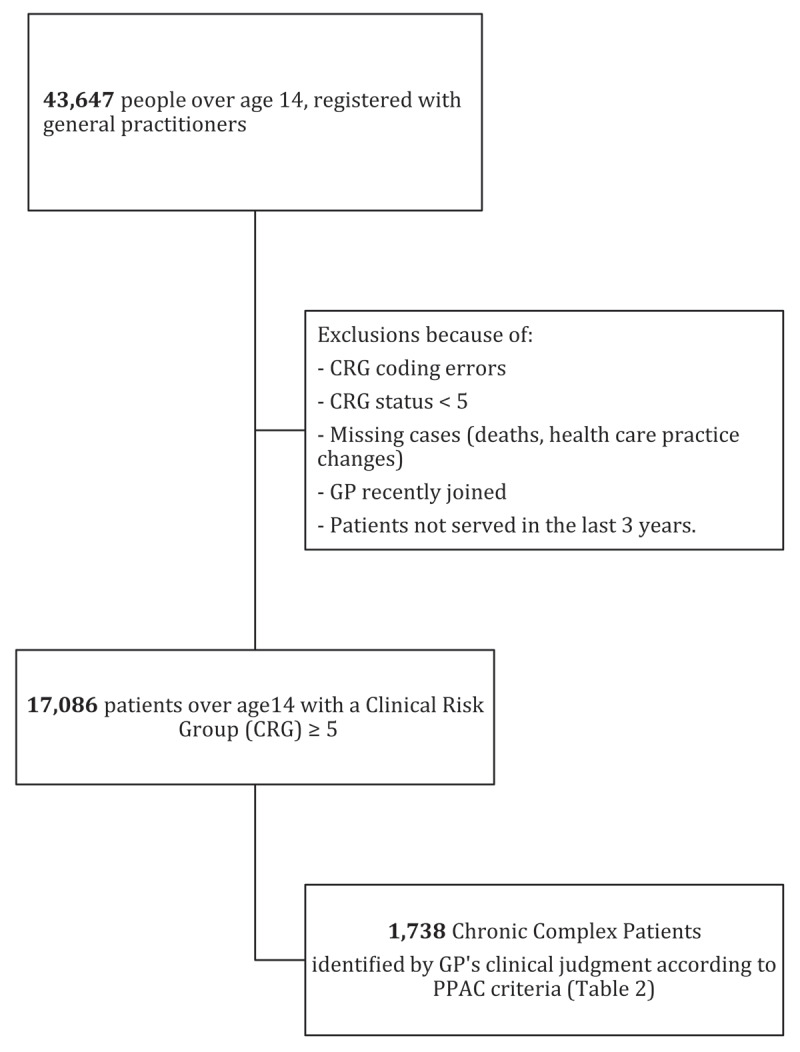
Sampling framework for data collection.

A list of all the patients assigned to each GP and who met the inclusion criteria described below was created from the ICS central information systems service. An administrative assistant created a list that would include patients over a three-month recruitment period. During this period of time, each GP independently reviewed his or her patients’ electronic medical records and completed the case report form for each patient. GPs who had recently joined (less than 1 year before the outset of the study) the primary care workforce were excluded from the study, due to their limited knowledge of the social and health backgrounds of patients in their lists. All GPs who participated in the study did so voluntarily, and participation and collection of study data were not remunerated. The sampling framework is depicted in ***Figure 1***.

### Defining concepts: Complex Chronic Patient (CCP) and advanced chronic disease and life-limited prognosis (MACA)

CCPs were defined according to the independent clinical judgment of their referent GPs. The list of domains of complexity designed by the PPAC [23] (***Table 2***) was adapted to the study and used as a reference. Among those patients identified as complex, a group was characterized as having advanced chronic disease and limited-life prognosis by the “surprise question”, included in the NECPAL-CCOMS-ICO © instrument [24]: “Would you be surprised if this patient died in the next 12 months?”

**Table 2:** Chronicity Prevention and Care Programme (PPAC) criteria to determine complexity.


COMPLEXITY CRITERIA	ANSWER

**Patient-dependent criteria**	

Multimorbidity (≥2 chronic diseases)	Yes/No/Don’t know

A single, severe chronic disease (including advanced frailty states)	Yes/No/Don’t know

A chronic progressive disease	Yes/No/Don’t know

High probability of undergoing decompensations with many symptoms and poor control	Yes/No/Don’t know

Patient with a variable, very dynamic evolution who needs continuous follow-up	Yes/No/Don’t know

High use of health services (emergency services, Primary care appointments)	Yes/No/Don’t know

Polypharmacy (≥5 medicines) and/or high cost of resources	Yes/No/Don’t know

Frail patients with functional loss, probability of acute deterioration (functional or cognitive) or new onset of geriatric syndromes	Yes/No/Don’t know

**Professional-dependent criteria**	

Need for multidisciplinary hospital management	Yes/No/Don’t know

Need to activate and manage access to different resources (often by priority routes)	Yes/No/Don’t know

Environment of special uncertainty in terms of decision-making or doubts in clinical management	Yes/No/Don’t know

**Social complexity**	

Patient with adverse psychosocial conditions	Yes/No/Don’t know

Patient whose management would benefit from integrated care strategies	Yes/No/Don’t know

Patient with relational problems	Yes/No/Don’t know

Patient with economic problems	Yes/No/Don’t know

Patient with loss of functional autonomy	Yes/No/Don’t know

**Other specific criteria**	

Patient with chronic neurological disease	Yes/No/Don’t know

Patient with severe mental disorder	Yes/No/Don’t know

Patient with dementia	Yes/No/Don’t know

Patient with intellectual disability	Yes/No/Don’t know

Elderly patient (≥75 years old)	Yes/No/Don’t know


### Variables

The following variables were collected: referent GP, age, gender, Clinical Risk Groups stratum (CRG) and Adjusted Morbidity Groups (GMA) stratum. PPAC criteria are shown in ***Table 2***. These criteria were established by the Department of Health on the basis of a systematic consensus of experts in the management of patients with clinical complexity.

The Clinical Risk Groups (CRG) system is a population classification system that uses inpatient diagnosis, ambulatory diagnosis, procedure codes, pharmaceutical data and functional health status to classify each individual into a hierarchically defined health status group. CRG classifies people into one of the following health states: 1) Healthy; 2) Significant acute illness; 3) Single or multiple minor chronic diseases; 4) Moderate chronic diseases; 5) Chronic dominant diseases; 6–7) Multiple chronic dominant diseases; 8) Advanced neoplastic disease; 9) Catastrophic diseases. Its purpose, among others, is to detect patients who require greater attention, to monitor prevalence rates of chronic diseases, to understand the patterns of use and consumption of services and to develop risk and price adjustment applications [25].

The Adjusted Morbidity Groups (GMA) classification is a new multimorbidity risk adjustment grouper developed by and adapted to the Spanish healthcare system. It was fully implemented into the workstation of primary care clinicians by May 2015. The GMA classifies individuals into unique and mutually exclusive groups by taking all the following into account: (i) type of disease; (ii) presence of multimorbidity; and (iii) case complexity. Four pyramidal strata are identified, with higher patient positions in the pyramid involving more complexity, more severity and a higher risk of mortality and hospital admission. The four pyramidal strata include: (i) GMA-1 or low-risk stratum, which includes 50% of the population with the lowest complexity level; (ii) GMA-2 or moderate risk stratum, which includes 30% of the population, with higher complexity than that found in the previous risk stratum; (iii) GMA-3 or high-risk stratum, which includes 15% of the population above the GMA-2 stratum; (iv) GMA-4 or very high risk stratum includes 5% of the population, with the highest complexity level [26]. The GMA classification has recently been validated and adopted by 13 of the 17 regional healthcare systems in Spain, covering 92% of the overall Spanish population (approximately 38 million citizens).

The risk of admission concerns the probability of urgent admission in the following twelve months adjusted for age, sex, socioeconomic status (MEDEA) and morbidity (GMA). A logistic regression (with either “urgent admission” or “not urgent admission” as the target variable) was applied, and different risk levels were assigned depending on the probability obtained.

### Statistical Methods

Descriptive statistics of the variables and cluster analysis were calculated using the SPSS software program (IBM; Chicago, IL; USA). Data were expressed as mean ± standard deviation for continuous variables, and as percentages when reporting categorical variables. U Mann-Whitney, Kruskal-Wallis and Chi-squared tests were used. Cluster analysis was applied to identify clinically relevant subgroup patterns of complexity. For this purpose, the SPSS two-step clustering (TSC) algorithm was employed. The TSC algorithm has unique features over other methods of clustering, such as an automatic procedure for calculating the optimal number of clusters, the ability to work with large data files and the ability to create models of clusters with both categorical and continuous variables. Naming of clusters is the most remarkable outcome in the study data.

We studied the robustness of the clusters in two steps. First, we selected 5 random samples of our data. After that, we applied the TSC algorithm to these samples. The results obtained in both cases were qualitatively and quantitatively similar.

### Confidentiality and ethical approval

The study was carried out in accordance with current legal regulations. This project has been approved by the Clinical Research Ethics Committee (CEIC) of the Institute for Research in Primary Care (IDIAP) Jordi Gol i Gorina (Barcelona) with reference number P15/119.

Patients’ informed consent was not a necessary requirement, as the only information required was that included in the shared electronic health records, from with the case report was filled in by the referent GP (neither interventions on patients nor the obtention of additional information were required in the study).

The study investigators were committed to comply with the Organic Law of Data Protection. As the database was anonymized, none of the data collected could be used to identify patients.

### Results

#### Sample characteristics

26 GP out of 28 contributed to the study. 1,738 patients were identified as CCPs (3.98% prevalence). 54.6% of the CCPs were female (mean age 75.16 ± 14.3 years). 61.6% of the patients identified as CCPs were over the age of 75. The distribution of CCPs in the primary health care centres is shown in ***Table 3***.

**Table 3:** Distribution of CCP patients in Primary Health Care centres according to gender, age (mean ± SD), risk of admission probability (mean ± SD) and GMA. In each of the centres, statistically significant differences were found between both genders in terms of age and risk of admission. Besides, statistically significant differences between centres were found in terms of the age and the risk of admission of the patients they served.


PRIMARY HEALTH CARE CENTRE		MALE	FEMALE	TOTAL	GMA/n/%

NORD	n (%)	269 (15.5%)	301 (17.3%)	**570 (32.8%)**	GMA 1 0; 0%

	Age (mean ± SD)	70,57 ± 15.43	74.8 ± 14.11	**72.81 ± 14.8**	GMA 2 36; 2.1%

	Risk of admission (mean ± SD)	18.51 ± 13.59	13.58 ± 9.77	**15.91 ± 11.98**	GMA 3 191; 11%

					GMA 4 343; 19.7%

CA N’ORIAC	n (%)	288 (16.6%)	314 (18.1%)	**602 (34.7%)**	GMA 1 5; 0.3%

	Age (mean ± SD)	75 ± 13.10	77.35 ± 13.57	**76.23 ± 13.4**	GMA 2 42; 2.4%

	Risk of admission (mean ± SD)	18.46 ± 11.9	14.13 ± 9.92	**16.20 ± 11.13**	GMA 3 196; 11.3%

					GMA 4 359; 20.7%

CONCORDIA	n (%)	249 (14.3%)	317 (18.2%)	**566 (32.5%)**	GMA 1 4; 0.2%

	Age (mean ± SD)	75.28 ± 14.23	77.29 ± 14.57	**76.4 ± 14.4**	GMA 2 41; 2.4%

	Risk of admission (mean ± SD)	18.7 ± 12.03	14.96 ± 10.48	**16.6 ± 11.33**	GMA 3 169; 9.7%

					GMA 4 352; 20.3%


Patients were characterized in three clusters, whose features are shown in ***Table 4***. Outcomes from the two-step cluster analysis are described and the percentage of positive responses in PPAC criteria are shown. Clusters were named according to the most remarkable findings in the data.

**Table 4:** Cluster characteristics. PPAC criteria: % of **positive** responses. The highest % in each variable is shown in bold.


VARIABLE	*CLUSTER 1* AMBULATORY LOW COST CCP	*CLUSTER 2* PSYCHOSOCIAL CCP	*CLUSTER 3* HIGH-NEED, HIGH-COST	*P VALUE*

**Patient characteristics**				

n (%)	**640** (36.8%)	**678** (39%)	**420** (24.2%)	

Age (mean ± SD)	79.5 ± 11,6	68.7 ± 16.5	78.9 ± 9.5	**.000**

Risk of admission (%) (mean ± SD)	16.2 ± 10,2	13 ± 10.4	21.4 ± 13	**.000**

Women (%)	54.60	50.90	56.70	**.145**

**Adjusted Morbidity Groups**				

GMA 1 (%)	0.5	**0.9**	0.0	**.000**

GMA 2 (%)	5.2	**12.1**	1.0	

GMA 3 (%)	31.4	**38.6**	22.1	

GMA 4 (%)	63.0	48.4	**76.9**	

**Chronicity Care and Prevention Program (PPAC) criteria**				

Multimorbidity	96.6	94.1	**99.3**	**.000**

1 chronic severe	27.7	**30.5**	26.7	**.56**

1 chronic progressive	71.4	74.6	**89.3**	**.000**

Decompensation, many symptoms and poor control	37.8	42.3	**92.9**	**.000**

Very dynamic evolution, continuous monitoring	19.2	19.3	**68.3**	**.000**

High use (Emergency services, Primary care appointments)	29.4	51.3	**78.3**	**.000**

Polipharmacy (≥5 medicines)	92.2	84.2	**98.6**	**.000**

Frailty+, acute deterioration, geriatric syndromes	58.3	53.7	**93.6**	**.000**

Age >75	**73.6**	43.2	73.1	**.000**

Neurological disease	19.7	17.6	**28.6**	**.000**

Severe mental disorder	4.4	**16.5**	8.8	**.000**

Dementia	23.1	13.6	**24**	**.000**

Psychic impairment	6.3	**10**	8.3	**.000**

Multidisciplinary hospital management	27.2	42.6	**70.7**	**.000**

Priority routes	6.7	17.3	**64.5**	**.000**

Uncertainty in decision-making, doubts in clinical management	10.9	40	**58.3**	**.000**

Advanced chronic disease and limited-life prognosis	15.3	14.6	**33.8**	**.000**

Adverse psychosocial conditions	13.3	**41.4**	32.4	**.000**

Integration benefit	30.1	50.6	**72.4**	**.000**

Relational problems	11.1	**28.6**	24	**.000**

Economic problems	0.6	**16.1**	8.8	**.000**

Loss of functional autonomy	50.6	44.4	**76**	**.000**


Three main clusters of CCPs were defined:

1. **Ambulatory Low Cost Chronic Complex Patients:** n = 640, 36.8% of the total. 54.5% of the patients in this cluster (mean age 79.5 ± 11.6) are female. The mean age of female patients in the cluster was 81.47 ± 10.9, whereas the mean age of male patients in the cluster was 77.23 ± 12 (p 0,000). Average risk of admission (16.2% ± 10.2) was found to be higher in male patients (18.6% [p 0,000]). 63% of the CCPs in this cluster belong to GMA 4. Most of them do not suffer decompensations, they are properly controlled and need neither continuous follow-up nor additional services besides primary care. More than half suffer from frailty and do not require multidisciplinary hospital management. Doubts and uncertainty in clinical decision-making arise in 11% of the patients in this cluster. Their psychosocial situation is good and, unlike the patients in the other two clusters, only 31% of them would benefit from social and health care integration. 50% of them have functional limitations in their ability to care for themselves or perform daily tasks. 50% of these complex patients are allocated in Ca N’Oriac Primary Care Centre.
2. **Psychosocial Chronic Complex Patients:** n = 678, 39% of the total. 51% of the patients in this cluster (mean age 68.7 ± 16.5) are female. The mean age of patients in this cluster was significantly lower than that of patients in the other two clusters (69.9 ± 16.7 for female patients and 67.3 ± 16.2 for male patients [p 0,011]). Average risk of admission (13.1% ± 10.4) was found to be higher in male patients (15.4% ± 11.7 [p 0,000]). 51% of the CCPs in this cluster belong to GMA 2 and 3. The youngest patients are found in this cluster, and there is less polipharmacy, multimorbidity, frailty, loss of functional autonomy and advanced chronic disease and limited-life prognosis than in the other two clusters. Neurological diseases and dementia are also less prevalent. However, this is the cluster with the highest proportion of severe mental disorders and psychic impairment. As opposed to what was found in the Ambulatory cluster, high use of care services (emergency services, primary care appointments) increases to nearly half of the patients. Doubts and uncertainty in clinical decision-making rises to 40%. The highest percentage of psychosocial deprivation was found in this cluster, but still it was the cluster where psychosocial deprivation remained unidentified to a greater extent. GPs working in the most deprived PHC (Cap Nord) contributed a higher proportion (46.2%) of complex patients to the Psychosocial Chronic Complex Patients Cluster than those GPs who worked in the other two practice settings.
3. **High-Need, High-Cost:** n = 420, 24.2% of the total. 56% of the patients in this cluster are female (mean age 78.9 ± 9.5). No significant differences between female and male patients (p 0.825) were found in this cluster. This is the cluster with the highest risk of admission (21.4% ± 13), which was higher in males (24.3% ± 13.6 [p 0.000]). 77% of the patients in this cluster are allocated in GMA 4, and 73% of them are over the age of 75. The vast majority of them present a variable, very dynamic evolution, need continuous follow-up and make heavy use of health services (emergency services, primary care appointments). Almost 100% of the patients in this cluster are polymedicated and are defined as frail patients with functional loss, probability of acute deterioration (either functional or cognitive) or new onset of geriatric syndromes. This is the cluster with the highest proportion of neurological diseases and dementia, and the one in which access to different resources must be activated and managed, often with priority routes. This is also the cluster where social and health care integration may be seen more clearly. Doubts and uncertainty in clinical decision-making increases to 60% of the patients. Loss of functional autonomy rises to 76% of them. The percentage of patients with advanced chronic disease and limited-life prognosis reaches 34%, the highest value in the three clusters.

**Figure 2:**
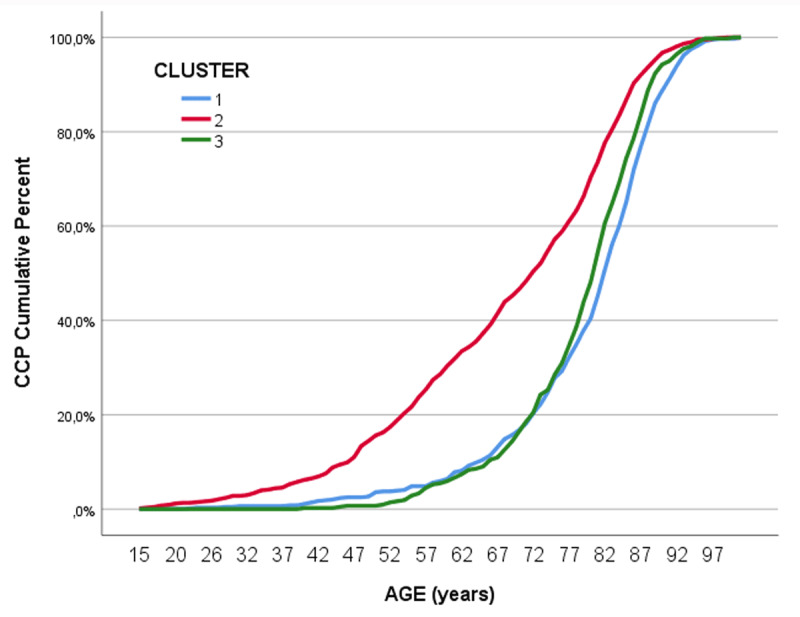
Prevalence of complexity by age and cluster.

CCPs in the Psychosocial cluster are more likely to be complex at all ages, and young and middle-aged patients in this cluster show rates of complexity that are equivalent to those of patients who are 20 years older and belong to the other two clusters (***Figure 2***).

## Discussion

Patients with complex health care needs account for approximately 50% of the total health care expenditure and receive care from multiple sources, although some of their health care and social needs are unmet [27]. To our knowledge, this is the first paper that applies empirical clustering to characterize sub-populations of CCPs from the GP’s perspective. Other studies have defined distinctive complex subgroups based on hierarchical conditions [28], condition-specific profiles and disease trajectories [29], clustering of household health expenditure and characteristics of high-cost families [30].

The main strengths of this research are its population-based approach and the involvement of Primary Care professionals in defining complex needs beyond multimorbidity and current risk adjustment. Spain has strong primary care services, and countries with such primary care systems (patient-centred care, access, longitudinality and care coordination with other providers) tend to perform better in chronic care management [31]. A panel of patients registered with a professional and its longitudinality [32], together with PPAC criteria, make it possible to understand the patients’ complex milieu of medical, mental health and social issues. In fact, the review of clinical records in our study increased baseline CCP prevalence to 4% when clinical judgement and PPAC criteria were taken into account together.

### There is more than meets the eye

**Figure 3:**
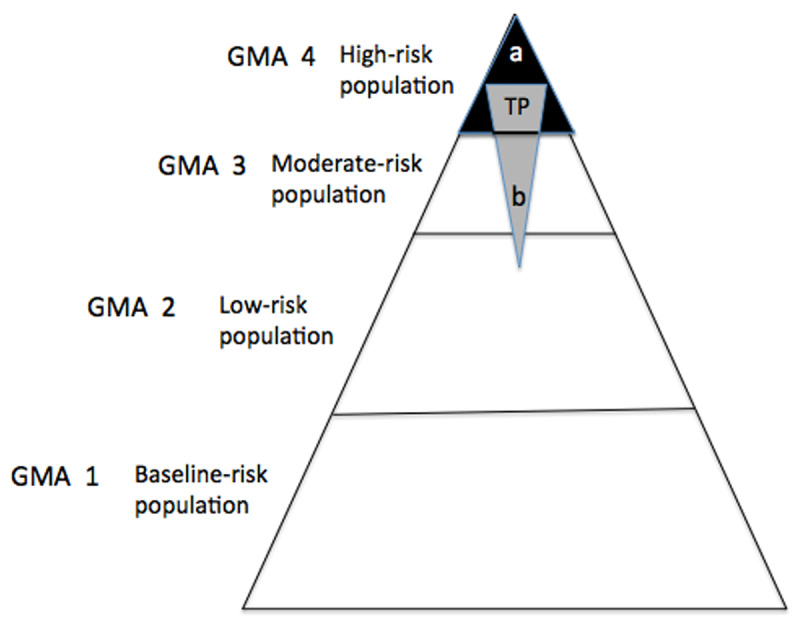
(i) Physicians’ perspective as the gold standard vs. GMA 4. **a** = False Positive, **TP** = True Positive, **b** = False Negative; (ii) GMA 4 as the gold standard vs. physicians’ perspective. **a** = False Negative, **TP** = True Positive, **b** = False Positive. In our data, 60.6% of CCPs are located in the GMA 4 stratum, 32% of CCPs are located in the GMA3 stratum and 7.4% of them are located in the GMA 2 stratum.

In business there is a 20/80 rule (20% of your customers generate 80% of the volume). In health care there is a 5/50 rule (5% of the patients account for half of all health expenditure). Health cost is an effective measure to characterize the complexity of any given patient and the use of healthcare resources. There is a high concentration and persistence of healthcare costs in a small part of the population, and healthcare costs and the burden of disease in the population are strongly correlated [33]. Our 4% prevalence of CCPs is not found exclusively among the top 5% of high-risk patients. According to the GMA pyramidal risk stratification, our sample would look like an inverted high-risk stratum that would include CCPs belonging not only to GMA 4, but also to GMA 3 and to GMA 2 (***Figure 3***). The latter would not be regarded as high-risk population, which further highlights the fact that complexity as defined by physicians moderately agrees with population grouping and risk stratification tools [16]. As a result, false positives and false negatives are obtained, depending on the model (if any) that is taken as a gold standard in order to define complexity. Weak predictive accuracy leads to a high percentage of false negatives (unjustified certainty, incorrectly classifying patients as low-risk or no-risk patients, delays in detection and worsening prognosis). False positives act as a warning to us in order to take opportunity costs into consideration; that is to say, minding the waste of time and resources devoted to prevention and interventions that could have been used in better alternatives and that generated unnecessary anxiety, overdiagnosis and overtreatment. Predictive models should not only identify high-risk patients, but also those patients who have a high risk of becoming high-cost patients in the future. If we bear in mind the definition of HNHC by Hayes et al. [8], “three or more chronic diseases and a functional limitation in their ability to care for themselves”, nearly half of our sample would not benefit from a redesign of tailored health care models. By combining clinical judgement and risk stratification, we might be talking about prevalence values that are over the 5% value reported in the literature.

### Psychosocial knowledge, a pending subject

We would like to underscore the high proportion of patients in the Psychosocial Chronic Complex Patients Cluster whose social concerns we know nothing about (***Table 5***). GPs are used to giving code numbers to biological issues. When compared with clinical diagnoses, social diagnoses (people with potential health hazards related to socioeconomic and psychosocial circumstances, Z55-Z65, ICD-10) are scarce in electronic health records [34]. At least one third of the patients who are served in general practices suffer from psychosocial problems that they perceive as influencing their present health status; general practitioners only identified between a fifth and a half of these problems, but continuity of care increased the likelihood of the care provider becoming aware of them [35]. In fact, whether care providers regard patients as challenging depends more heavily on the patients’ socio-economic status than on their medical problems alone, with interventions needed that exceed the scope of their medical expertise [36]. Our hypothesis is that there is a “social factor”, underappreciated by practitioners, that makes these patients complex. Efforts must be made in order to increase awareness on these concerns, and effective integrated (both clinical and social) care could help to prioritize them.

**Table 5:** PPAC social criteria. % of **Unknown** responses. The highest % in each variable is shown in bold.


VARIABLE	AMBULATORY LOW COST CCP CLUSTER	PSYCHOSOCIAL CCP CLUSTER	HIGH-NEED, HIGH-COST CLUSTER	*P VALUE*

Adverse psychosocial conditions	0.6	**51.2**	2.1	**.000**

Integration benefit	5.6	**39.8**	1.7	**.000**

Relational problems	1.9	**54.6**	6.2	**.000**

Economic problems	13.4	**75.4**	24.5	**.000**

Loss of functional autonomy	1.3	**9.4**	1.4	**.000**


Women predominated in all clusters, which accounts for the fact that women tend to live longer than men. And the longer a person lives, the higher the likelihood of functional limitations, which makes it more difficult to afford care for patients and their caregivers. That may be the reason, together with dementia and neurological diseases, of the high proportion of positive responses obtained when questions about the potential benefits of health and social care integration were asked in the High-Need, High-Cost Cluster. When asked about care management programs for high-risk patients, patients were much more worried about financial and caregiver issues than about their own health, which underscores the importance of the socioeconomic burdens and the need for programs to address these issues [36,37]. In a recent US survey, it was found that half of family caregivers who provide complex care perform medical and nursing tasks they often find difficult to learn and to use; besides, this increases their emotional and physical stress and makes them feel as if they had no choice [38].

### Complexity is not a static construct

This is a cross-sectional study, but a patient’s status changes over time, suggesting that complexity is not a static construct. After reviewing the literature, Figueroa et al. showed that 28% of patients who were high-cost persistently remained high-cost over the subsequent two years, while 72 % of them were transiently high-cost for 1 or 2 years [39]. In another study, few high-cost patients remained persistently high-cost over 4 years [28]. Persistently high-cost patients are generally older than episodic high-cost patients, and older ages are associated with persistently high costs [40]. In our study, some patients who belonged to the Ambulatory and Psychosocial clusters will irrevocably transition into the High-Need, High-Cost cluster as they age, multimorbidity increases and disability and/or frailty emerge, while others might no longer be complex as long as health and social needs are catered for or improved. Therefore, it is essential not only to identify the different profiles of needs in the cohort, but also to monitor them over time for a better and efficient integrated management.

### Complexity beyond multimorbidity

The Psychosocial Chronic Complex Patients Cluster – and to a lesser extent the High-Need, High-Cost cluster – include a higher percentage of mental health and social items. Reasons to focus on these items that clearly contribute to define complexity, and to avoid underestimating them, are described as follows. Inclusion of mental issues when defining clusters has to do with health care use and costs. In Canada, the average cost of mental health high-cost patients is higher than that of other high-cost patients. These patients are also younger, live in poorer neighbourhoods and have different patterns of health care utilization, so improving the quality of care for high-cost patients in general could not have the intended effect on mental health high-cost patients [41]. The psychosocial cluster readily resembles what Olivera et al. described. Besides, mental health diagnoses seem to be prevalent in cluster analysis for identifying sub-populations of complex patients [42]. Grant et al. suggested different complexity patterns depending on age, where mental health and substance abuse were identified as the main problems in younger complex patients, while decision-making and care coordination predominated in older age [16]. In a recent systematic review, depression was found to be the disease most commonly clustered, and it was paired with eight different diseases [43]. In our data, the Psychosocial cluster, which includes the youngest patients, shows as much multimorbidity as the other two clusters, but a higher prevalence of severe mental disease. For this cluster, no equivalent to the geriatrician exists, and for this reason a generalist service is more needed [1]. Family environment is also essential to define complexity and health expenditure, as similar lifestyles, unhealthy habits, beliefs relating to disease and health, low economic status and social deprivation are associated with high-cost users [40].

The prevalence pattern described in this study (***Figure 2***) has also been described with multimorbidity-mental health disorders and socioeconomic status pointing out to a consistent social gradient [1]. As M. Marmot said, “If the major determinants of health are social, so must be the remedies” [44].

### Advancing in the field of integrated care

Integrated care involves the coordination of several care providers from the three sectors (health care sector, social sector, third sector) [45]. Sharing medical records and patient lists in which eligible cases are discussed and care plans are designed and agreed upon with patients and family helps manage the Dependency Act, personal assistance for cleaning and support with basic daily activities, temporary respite care in case of increased caregiver burden or burnout, telecare, etc. Many CCPs in all clusters lose functional autonomy, as may be seen in ***Table 4***. When people become dependent, their homes become the center of their lives. Primary care professionals, along with social health workers, are in the best position to gain a first-hand impression on how people live: cleanliness, presence of caregivers, medication management, refrigerator and its contents, neighbours, social networks… That is to say, they get a glimpse of the patient’s socioeconomic characteristics beyond multimorbidity, and this allows care professionals to better understand user needs and to identify specific opportunities to improve safety and wellbeing. All this information should be available to all health care providers; actually, a comprehensive multidimensional assessment for all CCPs should be available, which would result in an individualized intervention care plan agreed upon with the patient and the family, in which the wishes of relatives and patients would be taken into account. In view of our results, we consider it necessary to redesign the individualized intervention care plan that we currently use to provide 24/7 health care to our CCPs, where medical issues prevail over social aspects and where patients’ needs may not match the items to choose from in the electronic shared health records.

Finally, in view of our results, we consider that CCPs will need a devoted professional (perhaps a GP) who is responsible for coordinating clinical-social care and for engaging CCPs in decisions about their own care. With the aging of the population, more GPs and geriatricians will be needed, and their clinical judgement, as well as validated stratification models, will be crucial to identify patients in greatest need of proactive and coordinated care. Clinical record integration is critical to foster communication between clinical and social providers and to identify and support informal caregivers. It is also crucial to redesign funding mechanisms and payment incentives to meet patients’ needs, sharing experiences and learning from successes and failures [46]. We would also like to underscore the fact that the surprise question used in our case report form performs, according to some authors, poorly to modestly as a predictive tool for death, with poorer results in non-cancer illness. Rather than being used to predict mortality, it should be used to promote a palliative approach in the identified patients [47]. A summary of an approach to care in view of our results is provided in ***Table 6***.

**Table 6:** A holistic approach to care that helps CCPs access the right services. +: Priority for patients with complex needs.


	AMBULATORY LOW COST CHRONIC COMPLEX PATIENTS CLUSTER	PSYCHOSOCIAL CHRONIC COMPLEX PATIENTS CLUSTER	HIGH-NEED, HIGH-COST CLUSTER

**Preventive approach**	++	+++	+

**Palliative approach**	+	+	+++

**Self-care**	+++	++	+

**Collaborative health care**	+	+++	+++

**Integrated care (social)**	+	+++	+++


## Limitations

The use of electronic health records in our study could be limited by missing, incorrect or unverified data. The multimorbidity prevalence values to be found are influenced by the quality of electronic health data, by conditions that are not included in formal disease classifications (such as chronic pain) and by the introduction of risk factors (obesity, lipid disorders) as diseases. Given that only patients in the Clinical Risk Group health state ≥ 5 could be included in the study, patients with a single or with multiple minor chronic diseases were not taken into account. In order to prevent inter-observer variability, GPs were trained on how to use the case report form.

Complexity from a primary care perspective may be influenced by other providers (different standards in terms of hospital admission, not related to morbidity, and admission rates due to bed supply-availability rather than care needs), and by several factors such as the resources available within the health area, vocational training, professionalism, and coordination with other health workforces such as specialists and social services [48].

In the absence of a gold standard to describe patient complexity, the results should be understood from the perspective of Primary Care workforces. We rely on these professionals’ knowledge in order to determine how the social and economic background of chronic patients can influence health-related complexity. We focus on the GP perspective, while other research has developed a definition of complexity that focused on the patients’ functional status: the balance between the workload of demands on a patient and the patient’s capacity to address such demands [13]. The results may not be extrapolated to other Primary Care scenarios, especially those where panels and patient-related longitudinality are discouraged.

## Conclusions

Efforts to characterize chronic complex patients are needed to achieve better care and assist patients to make healthcare decisions that are aligned with their goals. Such efforts often rely on a priori assumptions (multimorbidity, previous health care costs), and this may hide underlying complexities, particularly among low-income population and individuals with unmet social needs. Combining GPs’ perspective with population grouping and risk stratification tools could help to identify more accurately those CCPs who would be most likely to benefit from improvements, thus increasing effectiveness and efficiency. Different patterns of use and needs may be observed when recognizable subgroups of patients are defined among complex chronic patients on the basis of clinical and social criteria from the primary care physician’s perspective. This has important implications for providing continuity of care, coordinated services, clinical management and decision-making.

Even though more research is needed, this valuable information can be used to reduce reactive and fragmented care, to better target care improvements and integration strategies and to reduce expenditure over time, in order to design effective interventions and proactive approaches targeted to the patients who are at risk and who would most likely benefit from such interventions and approaches.
